# Candida auris Biofilm Colonization on Skin Niche Conditions

**DOI:** 10.1128/mSphere.00972-19

**Published:** 2020-01-22

**Authors:** Priya Uppuluri

**Affiliations:** a Division of Infectious Disease, The Lundquist Institute at Harbor—University of California, Los Angeles (UCLA) Medical Center, Torrance, California, USA; b David Geffen School of Medicine, University of California, Los Angeles, Los Angeles, California, USA

**Keywords:** *Candida auris*, biofilm, skin, porcine, sweat, nosocomial, fungi

## Abstract

Candida auris, an emerging multidrug-resistant yeast, has recently been associated with outbreaks of invasive infections in health care facilities worldwide. Its success as a nosocomial pathogen lies in its capability to sustain for prolonged periods in the intensive care unit (ICU), adeptly colonize skin, and spread among patients. Little is known of the mechanism behind the predilection of C. auris for skin or the extent of its resilience on it. Now, M. V. Horton, C. J. Johnson, J. F. Kernien, T. D. Patel, et al. (mSphere 5:e00910-19, 2020, https://doi.org/10.1128/mSphere.00910-19) demonstrate that in synthetic sweat medium designed to mimic axillary skin conditions, C. auris can grow into multilayers of cells called biofilms that can resist desiccation. C. auris’ propensity to form biofilms was further elaborated using a novel *ex vivo* porcine skin model of skin colonization. These studies provide early evidence that C. auris biofilm cells persisting on skin could serve as source of continuing outbreaks in health care facilities. Interventions blocking C. auris biofilm growth on skin will help control the spread of this pathogen.

## Abstract (precis)

Candida auris, an emerging multidrug-resistant yeast, has recently been associated with outbreaks of invasive infections in health care facilities worldwide. Its success as a nosocomial pathogen lies in its capability to sustain for prolonged periods in the intensive care unit (ICU), adeptly colonize skin, and spread among patients. Little is known of the mechanism behind the predilection of C. auris for skin or the extent of its resilience on it. Now, M. V. Horton, C. J. Johnson, J. F.

## COMMENTARY

Candida auris is an emerging multidrug-resistant fungal pathogen that can cause invasive infections predominantly in immunocompromised hospitalized patients (1, 2). First isolated in 2009 from the ear canal of a Japanese patient (3), C. auris has been reported to cause disseminated diseases associated with mortality rates as high as 60% (4). Of concern is that only within the past decade, C. auris pathogenic isolates have appeared in nine countries and four continents, including the Unites States (1, 5–7). In fact, a report from a referral hospital in sub-Saharan Africa revealed that incidence of C. auris candidemia surpassed that of cases of Candida albicans, the most successful human fungal pathogen (5). Given the simultaneous worldwide outbreak of C. auris, the Centers for Disease Control and Prevention has designated this fungal pathogen a global public threat and has recently published an alert for health care facilities to surveil and report new cases of C. auris patients (8).

Unlike other pathogenic *Candida* species such as C. albicans that predominantly exist as a part of the normal flora of humans, C. auris can persist and thrive in the environment of health care facilities (6, 9). Because of this, C. auris possesses characteristics representative of a pathogen that can cause nosocomial infections (10), such as its (i) propensity to cause outbreaks likely due to spread by horizontal transmission, (ii) ability to cause life-threatening disease in immunocompromised patients, and (iii) multidrug resistance profile. Indeed, C. auris transmission occurs primarily among patients with a prolonged hospital stay.

Recent reports have shown that C. auris can form a community of cells called biofilms on surfaces recovered from hospital rooms during outbreaks (9, 11). However, these biofilms on inanimate surfaces are found to be rudimentary and weaker than robust biofilms typically produced by C. albicans (11, 12). The extent and mechanism of colonization of C. auris on human skin has not been explored. Given that C. auris can spread like wildfire between hospital rooms and health care facilities, *via* human contact, it is important to understand how C. auris perseveres on skin.

To understand the mechanism of skin colonization, Horton and colleagues analyzed growth of C. auris in synthetic sweat medium designed to mimic axillary skin conditions *in vitro* (13) Under these conditions, C. auris grew as a multilayer biofilm composed of yeast cells, with cellular burden intriguingly 10-times greater than that formed by C. albicans, a fungus that is otherwise adept at robust biofilm growth. Conversely, as published previously, C. auris developed only a thin monolayer of cells in standard laboratory medium, indicating that components in the synthetic sweat medium such as high salinity, fatty acids, etc. may provide C. auris with a growth advantage over C. albicans (14). Indeed, this finding was further evidenced when C. auris (but not C. albicans) biofilms could persist under stress conditions representing desiccation. In concentrated sweat medium (mimicking evaporated sweat), C. auris biofilms continued to thrive for 14 days, while C. albicans biofilm cells succumbed to this dehydrated environment only within the first week of growth. It is no surprise, then, that robust biofilm growth and ability to withstand dry surfaces make C. auris a successful environmental pathogen.

To understand the interaction of C. auris directly with skin, Horton et al. (13) simulated C. auris growth on an *ex vivo* porcine skin model. This novel skin model shares several characteristics with human skin and thus is a clinically relevant model (15, 16). Additionally, this model specifically supports the scenario of fungal colonization rather than invasion, making it ideal to study persistence rather than infection. As witnessed *in vitro*, C. auris developed multiple aggregates and robust biofilms on porcine skin, while C. albicans showed little fungal growth. Together, the studies suggest that part of the reason that makes C. auris such a successful pathogen is its ability to adeptly colonize skin niches and withstand environmental stresses. Certainly, recent isolated reports on outbreak surveillance from hospitals in the United States have implicated skin colonization by C. auris as one of the risk factors for nosocomial infections (9, 14). The study by Horton et al. (13) reinforces this finding by showing that a biofilm architecture not only serves as a high-burden reservoir of cells that can be shed intermittently upon contact but also provides C. auris with an enhanced ability to withstand the vagaries of environmental stresses. The latter advantage is not surprising, considering that a biofilm phenotype is known to protect microbial cells from a number of stresses, including immune cell assault and antimicrobial drugs (17).

In summary, Horton et al. (13) have taken the first steps to understand the interaction of C. auris with the skin milieu by designing inventive *in vitro* assays and *ex vivo* experimental models of axillary skin colonization. These models are clinically relevant, considering that reusable axillary probes have been linked to C. auris outbreaks (18). A more serious implication of skin colonization by C. auris biofilms is perhaps intravenous catheter contamination by skin puncture, which can carry the pathogen directly into the bloodstream. Indeed, the presence of indwelling medical devices is a primary risk factor for persistent candidemia by C. auris (1, 4, 6). An important future direction of this research would be harnessing the experimental models of colonization to identify molecular pathways underlying adherence of C. auris to the skin and to discover genetic determinants of biofilm growth. This knowledge will be critical for the thoughtful development of new interventional strategies against C. auris biofilm growth and persistence on skin.
